# Romidepsin suppresses monosodium urate crystal-induced cytokine production through upregulation of suppressor of cytokine signaling 1 expression

**DOI:** 10.1186/s13075-019-1834-x

**Published:** 2019-02-06

**Authors:** M. C. P. Cleophas, T. O. Crişan, V. Klück, N. Hoogerbrugge, R. T. Netea-Maier, C. A. Dinarello, M. G. Netea, L. A. B. Joosten

**Affiliations:** 1 Department of Internal Medicine, Radboud University Medical Center, Nijmegen, the Netherlands; 2 Radboud Institute for Molecular Life Sciences (RIMLS), Radboud University Medical Center, Nijmegen, the Netherlands; 3 Department of Medical Genetics, Iuliu Haţieganu University of Medicine and Pharmacy, Cluj-Napoca, Romania; 4 Division of Infectious Diseases, Department of Medicine, University of Colorado, Denver, Aurora, CO 80045 USA; 5 Department of Human Genetics, Radboud university medical center, Nijmegen, the Netherlands; 6 Department of Internal Medicine, Division of Endocrinology, Radboud University Medical Center, Nijmegen, the Netherlands; 7 Department for Genomics & Immunoregulation, Life and Medical Sciences Institute (LIMES), University of Bonn, 53115 Bonn, Germany

**Keywords:** Gout, Cytokines, Inflammation, HDAC

## Abstract

**Background:**

Acute gouty arthritis currently is the most common form of inflammatory arthritis in developed countries. Treatment is still suboptimal. Dosage of urate-lowering therapy is often too low to reach target urate levels, and adherence to therapy is poor. In this study, we therefore explore a new treatment option to limit inflammation in acute gout: specific histone deacetylase (HDAC) inhibition.

**Methods:**

Peripheral blood mononuclear cells (PBMCs) were cultured with a combination of monosodium urate crystals (MSU) and palmitic acid (C16.0) in order to activate the NLRP3 inflammasome and induce IL-1β production. HDAC inhibitors and other compounds were added beforehand with a 1-h pre-incubation period.

**Results:**

The HDAC1/2 inhibitor romidepsin was most potent in lowering C16.0+MSU-induced IL-1β production compared to other specific class I HDAC inhibitors. At 10 nM, romidepsin decreased IL-1β, IL-1Ra, IL-6, and IL-8 production. IL-1β mRNA was significantly decreased at 25 nM. Although romidepsin increased *PTEN* expression, PBMCs from patients with germline mutations in *PTEN* still responded well to romidepsin. Romidepsin also increased *SOCS1* expression and blocked STAT1 and STAT3 activation. Furthermore, experiments with bortezomib showed that blocking the proteasome reverses the cytokine suppression by romidepsin.

**Conclusions:**

Our results show that romidepsin is a very potent inhibitor of C16.0+MSU-induced cytokines in vitro. Romidepsin upregulated transcription of *SOCS1,* which was shown to directly target inflammatory signaling molecules for proteasomal degradation. Inhibiting the proteasome therefore reversed the cytokine-suppressive effects of romidepsin. HDAC1/2 dual inhibition could therefore be a highly potent new treatment option for acute gout, although safety has to be determined in vivo.

**Electronic supplementary material:**

The online version of this article (10.1186/s13075-019-1834-x) contains supplementary material, which is available to authorized users.

## Background

Acute gouty arthritis is an agonizingly painful and debilitating disease and is currently the most common form of inflammatory arthritis in developed countries. Its prevalence has been rising steadily over the past decades [1, 2], and it is currently estimated to affect 1–4% of the general population in most countries in Europe and in the USA [3–6].

The most important risk factor for the development of acute gout is hyperuricemia. Urate is a breakdown product of purine metabolism, and its levels can be elevated in the blood due to several causes, including genetic variations in urate transporter genes or a purine-rich diet. When urate reaches supersaturation in the biologic fluids or tissues, it precipitates as monosodium urate crystals (MSU) [7]. Interestingly however, the majority of hyperuricemic individuals stay asymptomatic throughout their life [2], and some patients have normal urate levels during an acute gout attack [8], indicating that induction of clinical symptoms by MSU crystals is a process that is dependent on many factors.

Nevertheless, the main prerequisite for the development of acute gout are MSU crystal deposits in the joint. It has been known for about a decade now that MSU crystals trigger inflammatory arthritis through activation of the nucleotide-binding domain and leucine-rich repeat-containing family, pyrin domain-containing 3 (NLRP3) inflammasome [9]. Assembly of this protein scaffold activates procaspase-1, which in turn can cleave inactive pro-IL-1β into its bioactive form. However, for the initial production of pro-IL-1β, an additional signal is required, such as IL-1β binding to the IL-1 receptor, or Toll-like receptor (TLR) ligands. Saturated long-chain fatty acids, such as palmitic acid (C16.0) and stearic acid (C18.0), can also provide this first signal and synergize with MSU crystals to produce large quantities of active IL-1β [10, 11].

Despite the elaborate knowledge on the pathophysiology of gout and the fact that it is curable, treatment is still suboptimal in many cases. Dosage of urate-lowering therapy is often too low to reach target urate levels, and adherence to therapy is poor among gout patients [12]. Furthermore, many of the comorbidities associated with gout actually result in contraindications for the currently available anti-inflammatory medication used in acute gout [13]. Biologics (recombinant antibodies) blocking IL-1β are advised in case NSAIDs and colchicine are contraindicated [12], but they are very costly [14]. In the current study, we investigated the potential of specific histone deacetylase (HDAC) inhibitors to suppress MSU-dependent cytokine production.

HDACs are enzymes capable of removing acetyl groups from protein lysine residues. With this ability, they can alter gene expression (when deacetylating histone lysines) or affect the function of non-histone proteins such as transcription factors or other enzymes [15]. The HDAC enzymes are divided into four classes based on phylogenetic analysis: class I yeast Rpd3-like deacetylases (HDAC1, HDAC2, HDAC3, and HDAC8), class II yeast Hda1-like deacetylases (HDAC4, HDAC5, HDAC6, HDAC7, HDAC9, HDAC10), class III yeast Sir2-like sirtuins (SIRT1, HDAC2, HDAC3, HDAC4, HDAC5, HDAC6, HDAC7), and class IV deacetylase (HDAC11). The classical HDACs of classes I, II, and IV and the sirtuins of class III make up two distinct families [16, 17]. Together, these enzymes can elicit widespread effects through changing gene transcription and posttranscriptional modification of proteins.

Next to several naturally occurring HDAC inhibitors, such as the short-chain fatty acid butyrate and *Streptomyces*-derived trichostatin A [16], a range of synthetic inhibitors has been developed, with the primary aim of treating malignancies. The rationale behind this use is the HDAC-mediated suppression of genes involved in apoptosis, cell cycle arrest, and tumor suppression [18]. However, in recent years, research has revealed a potential use of HDAC inhibitors to treat inflammatory diseases.

In the current study, we attempt to pinpoint the individual HDACs that could play a role in acute gouty arthritis by assessing the effects of specific HDAC inhibitors on C16.0+MSU-induced cytokine production by human PBMCs in vitro.

## Materials and methods

### Reagents and inhibitors

MSU crystals were produced in-house from urate (Sigma) and sodium hydroxide (Merck) as described previously [11]. Palmitic acid (Sigma) dissolved in 100% ethanol and human albumin (Albuman 200 g/L, Sanquin, Amsterdam) were conjugated as described previously [11]. Romidepsin, entinostat, santacruzamate A, RGFP966, and bortezomib were purchased from Selleckchem. Etomoxir was purchased from Sigma. HDAC6 inhibitor ITF3107 was kindly provided by Italfarmaco SpA, Milan, Italy.

### PBMC stimulation experiments

Venous blood was drawn from healthy donors or Cowden syndrome patients. PBMCs were isolated by means of Ficoll-Paque (GE Healthcare) density gradient centrifugation. Cells were plated at 0.5 × 10^6^ cells per well in a U-bottom 96-well plate with Dutch Modified RPMI 1640 medium (Life Technologies) supplemented with 50 μg/mL gentamycin (Centrafarm), 2 mM GlutaMAX, and 1 mM pyruvate (Life Technologies). Cytokine responses to mimic gout in vitro were induced by adding a combination of 300 μg/mL MSU crystals and 50 μM C16.0. For all experiments, the cells were cultured for 24 h.

### Cytokine measurements

Commercially available ELISA kits (R&D Systems) were purchased for IL-1β, IL-1Ra, IL-6, IL-8, and IL-10 which were used according to the manufacturer’s protocol. Intracellular IL-1β and IL-1Ra were determined in the supernatant of a cell lysate. Cells were lysed using 0.5% Triton X-100 (Sigma).

### Quantitative PCR

RNA was isolated using a phase separation method with TRIzol reagent (Life Technologies) and chloroform (Merck) in a 5:1 ratio. Subsequently, the RNA was precipitated with 2-propanol (Merck). Reverse transcription into cDNA was performed using iScript cDNA synthesis kit (Bio-Rad). Quantitative PCR was done using a SYBR Green PCR master mix (Life Technologies).

### Flow cytometry

Cells were fixed and permeabilized with FIX & PERM Cell Permeabilization Kit (Invitrogen) and subsequently stained with CD45-KO (Beckman Coulter) and p-STAT1-PE or p-STAT3-PE (eBioscience). Samples were measured on the Beckman Coulter CytoFLEX. During analysis, cells were gated on being CD45^+^ to eliminate debris. Gating on monocyte and lymphocyte populations was done in the forward versus side scatter plot.

### Statistics

All figure panels include ≥ 6 donors divided over at least two experiments. Conditions were compared to C16.0+MSU alone (unless stated otherwise in the figures) with a Wilcoxon signed rank test.

## Results

### Effects of specific HDAC inhibitors on decreasing C16.0+MSU-induced IL-1β

**Fig. 1:**
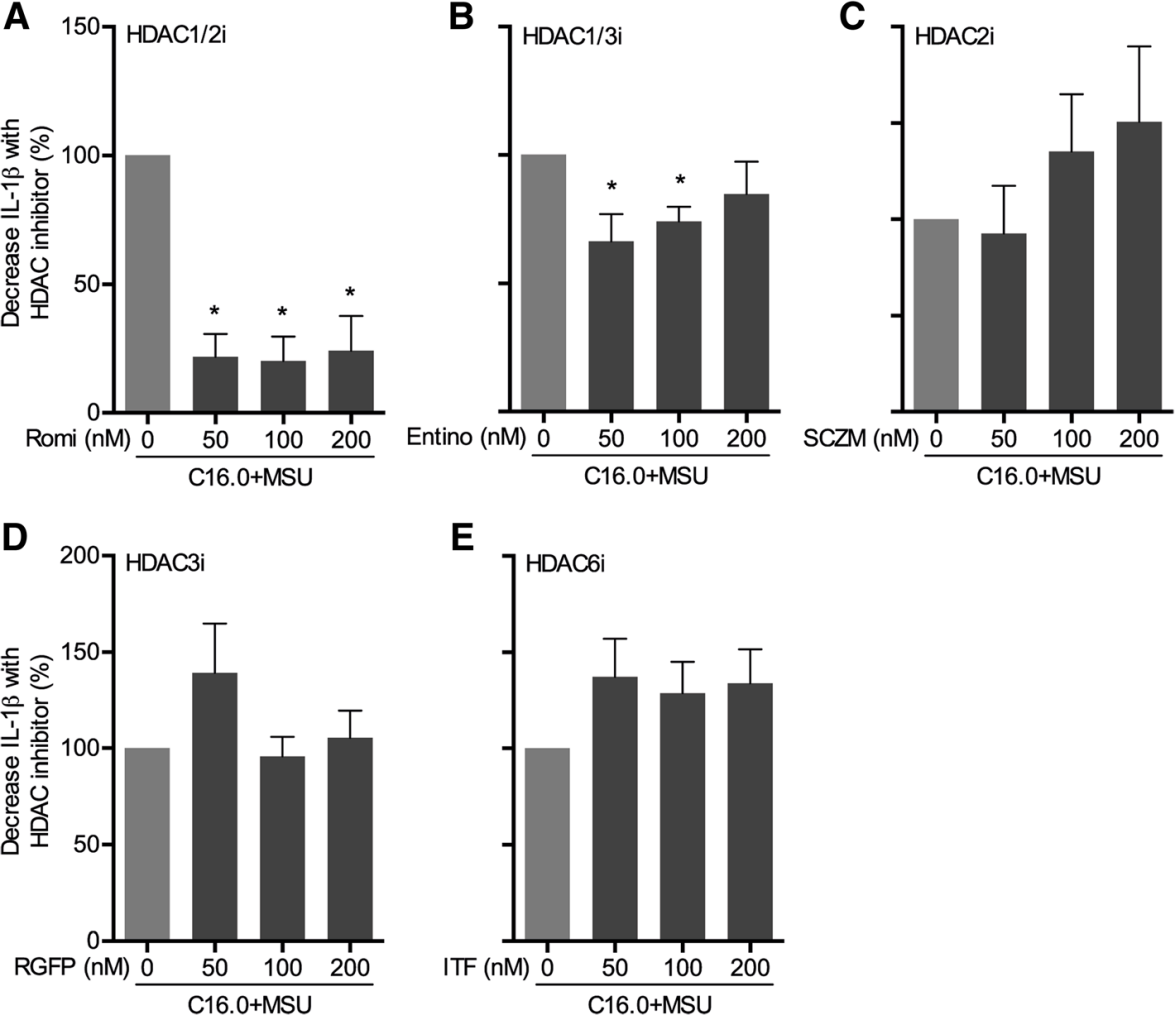
Screening of different class I HDAC inhibitors for their suppressive effect on C16.0+MSU-induced IL-1β. Freshly isolated PBMCs from healthy volunteers were pre-incubated for 1 h with different HDAC inhibitors: **a** romidepsin (Romi, HDAC1/2 inhibitor), **b** entinostat (Entino, HDAC1/3 inhibitor), **c** Santacruzamate A (SCZM, HDAC2 inhibitor), **d** RGFP966 (RGFP, HDAC3 inhibitor), and **e** ITF3107 (ITF, HDAC6 inhibitor). IL-1β production was induced by adding a combination of 50 μM palmitic acid (C16.0) and 300 μg/mL monosodium urate crystals (MSU) for 24 h. Data are represented as percentage change compared to the IL-1β production with C16.0+MSU alone

Because the first production of IL-1β is of crucial importance for the development of acute gouty arthritis, we tested the ability of several specific HDAC class I inhibitors to decrease its production in PBMCs in response to a combination of C16.0 and MSU (Fig. 1). In a previous study, we have shown that broad class I HDAC inhibition suppresses C16.0+MSU-induced cytokine production, but specific HDAC8 inhibition did not have a cytokine-suppressive effect [11]. Here, both romidepsin and entinostat significantly reduced C16.0+MSU-induced IL-1β production (Fig. 1a, b). However, the effect of romidepsin was significantly more potent, decreasing up to 80% of the IL-1β production, whereas entinostat inhibited up to 34%. The other specific HDAC inhibitors did not have any effect on C16.0+MSU-induced IL-1β production.

### HDAC1/2 inhibitor romidepsin strongly inhibited C16.0+MSU-induced cytokines

**Fig. 2:**
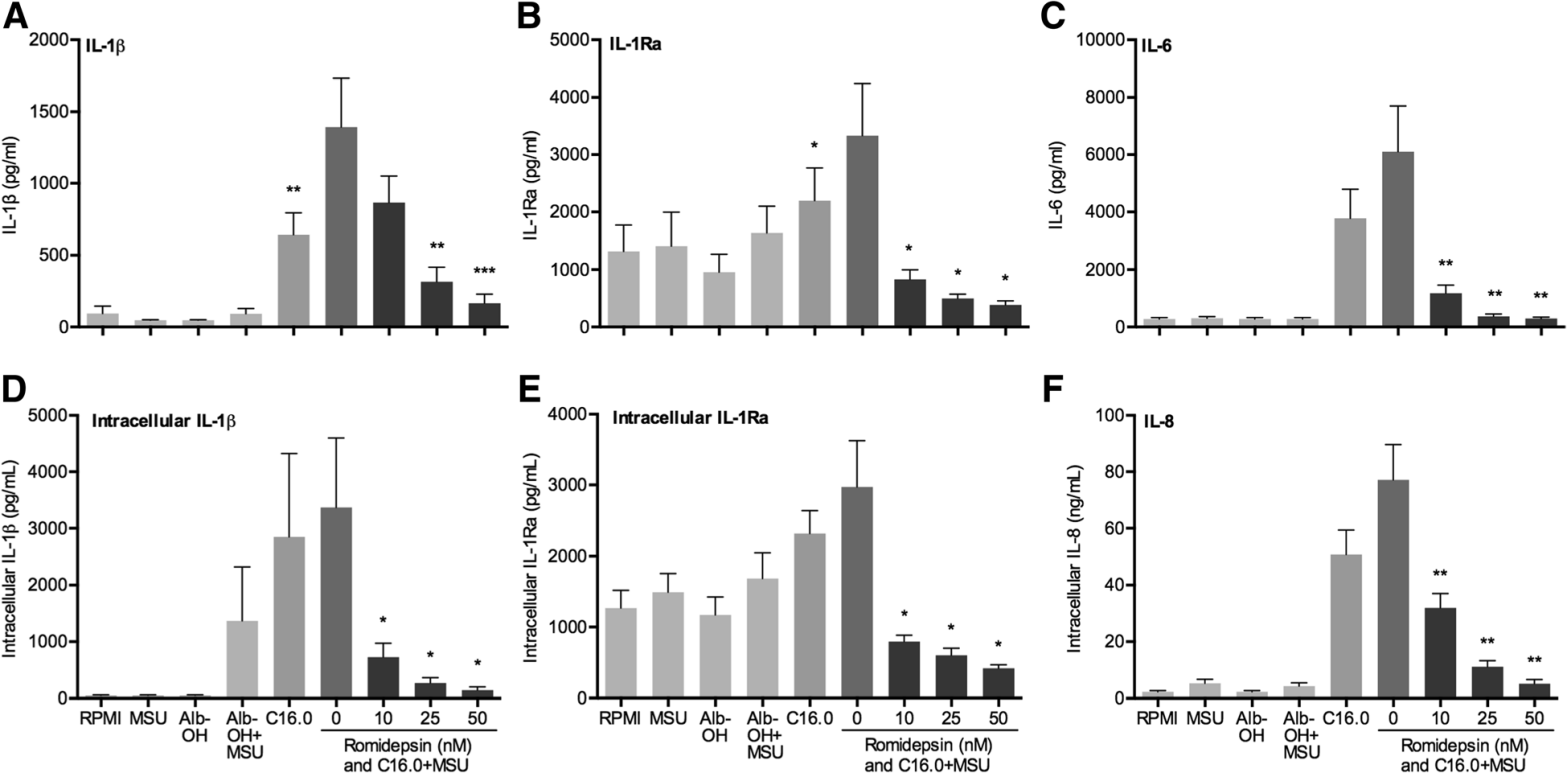
Suppressive effects of romidepsin on C16.0+MSU-induced cytokine production in human PBMCs. Freshly isolated PBMCs were pre-incubated with different concentrations of romidepsin for 1 h, after which cytokine production was induced via addition of a combination of 50 μM palmitic acid (C16.0) and 300 μg/mL monosodium urate crystals (MSU). The supernatant was collected for extracellular cytokines (**a**–**c**, **f**). Cells were lysed with 0.5% Triton X-100, and subsequently, the supernatant of the lysate was collected for measurement intracellular cytokines (**d**–**e**)

Due to the potent cytokine-suppressive effects observed with HDAC1/2 inhibitor romidepsin, we decided to further examine its effects. As shown in Fig. 2, already at a dose as low as 10 nM romidepsin significantly inhibited IL-1β, IL-6, IL-8, and IL-1Ra production in response to C16.0+MSU stimulation in PBMCs. In addition, intracellular IL-1β levels were decreased significantly as well (Fig. 2d). C16.0 alone only slightly increased the production of anti-inflammatory IL-10 (Additional file 1: Figure S1), and there is an insignificant trend towards IL-10 decrease when romidepsin is added.

### Effects of HDAC1/2 inhibition on cell viability and transcriptional levels

**Fig. 3:**
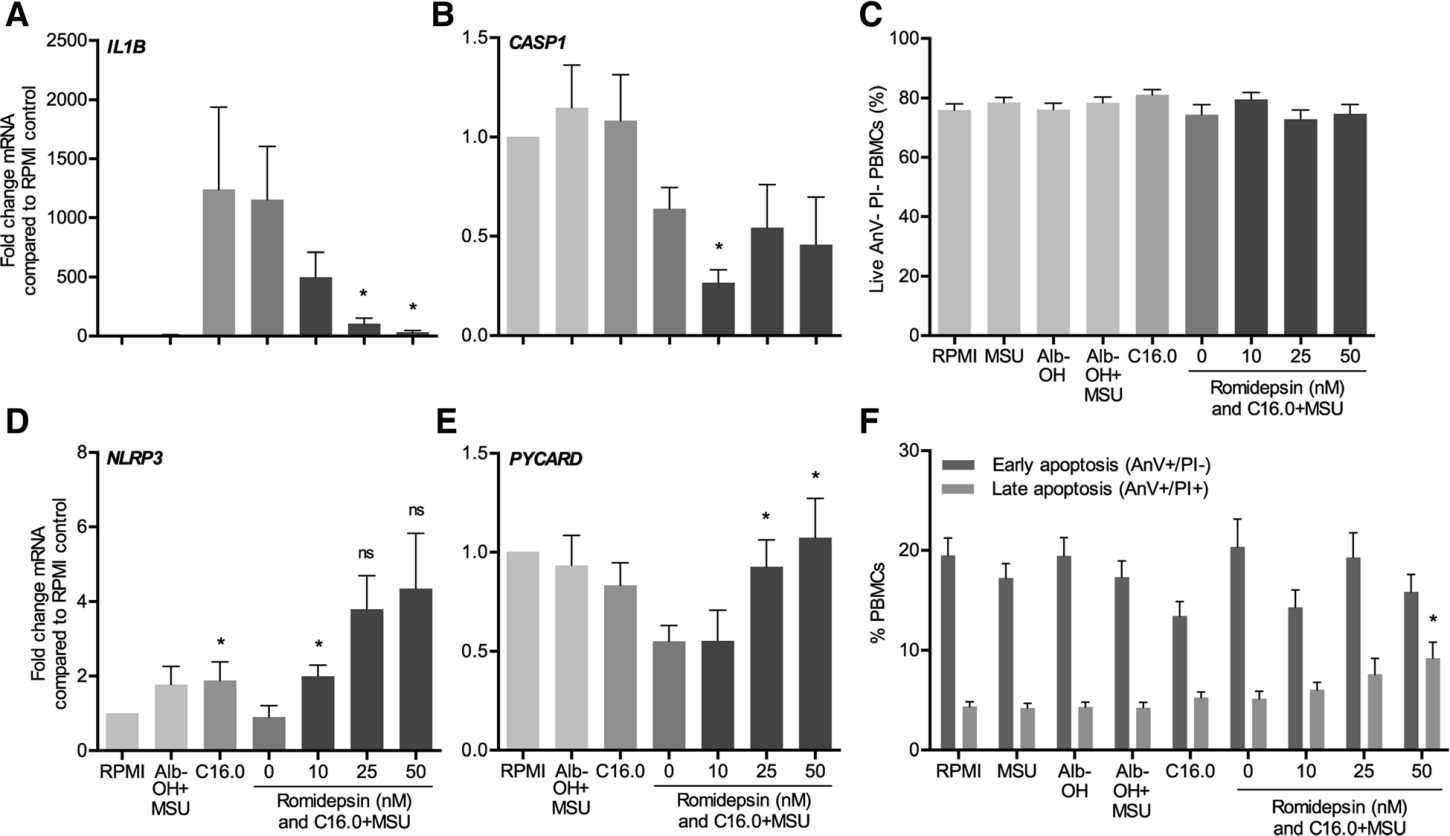
Effects of romidepsin on cell viability and transcription of IL-1β and NLRP3 inflammasome components. Freshly isolated PBMCs from healthy donors were pre-incubated with different concentrations romidepsin for 1 h, after which a combination of 50 μM palmitic acid (C16.0) and 300 μg/mL monosodium urate crystals (MSU) was added to induce cytokine production. After 24 h of culture, mRNA was measured for *IL1B* (**a**), *CASP1* (**b**), *NLRP3* (**d**), and *PYCARD* (**e**). Percentages of Annexin V^+^ and PI^+^ were measured by flow cytometry to determine cell viability (**c**, **f**)

After determining that romidepsin effectively inhibits C16.0+MSU-induced cytokine production, we also examined the transcription of IL-1β and inflammasome-related genes. In line with previous observations [11], we see that the combination of C16.0+MSU induces a similar amount of IL-1ß as is induced by C16.0 alone (Fig. 3a). This supports the current theory of the two signals that are required to induce active IL-1ß, as is described in the introduction. Whereas MSU crystals induce NLRP3 assembly and caspase-1 activation, it has no effect on transcription of pro-IL-1ß. This signal is provided by C16.0.

In Fig. 3a, we show that IL-1β mRNA transcription was induced dramatically by C16.0-stimulation and was almost brought back to baseline levels by romidepsin. mRNA levels of NLRP3 inflammasome components were not as consistently modified by romidepsin. The lowest concentration of romidepsin of 10 nM significantly decreased *CASP1* and increases *NLRP3* transcription (Fig. 3b, d). The higher concentrations of romidepsin increased transcription of adaptor protein *ASC (PYCARD)* in comparison to C16.0+MSU alone, but not in comparison to the medium control (Fig. 3e). Following the drastic decreases in cytokine production upon addition of romidepsin, we wanted to ensure that cells were still viable after incubation by means of flow cytometry with Annexin V (AnV) and propidium iodide (PI) staining. Neither stimulation with C16.0+MSU nor addition of romidepsin affected the percentage of live (Anv and PI negative) cells (Fig. 3c). When looking at the stratification of early (AnV^+^PI^−^) and late (Anv^+^PI^+^) apoptotic cells, only the percentage of late apoptotic cells was increased with 50 nM romidepsin (Fig. 3f).

### Cytokine-suppressive effects of romidepsin independent of PTEN mRNA upregulation

**Fig. 4:**
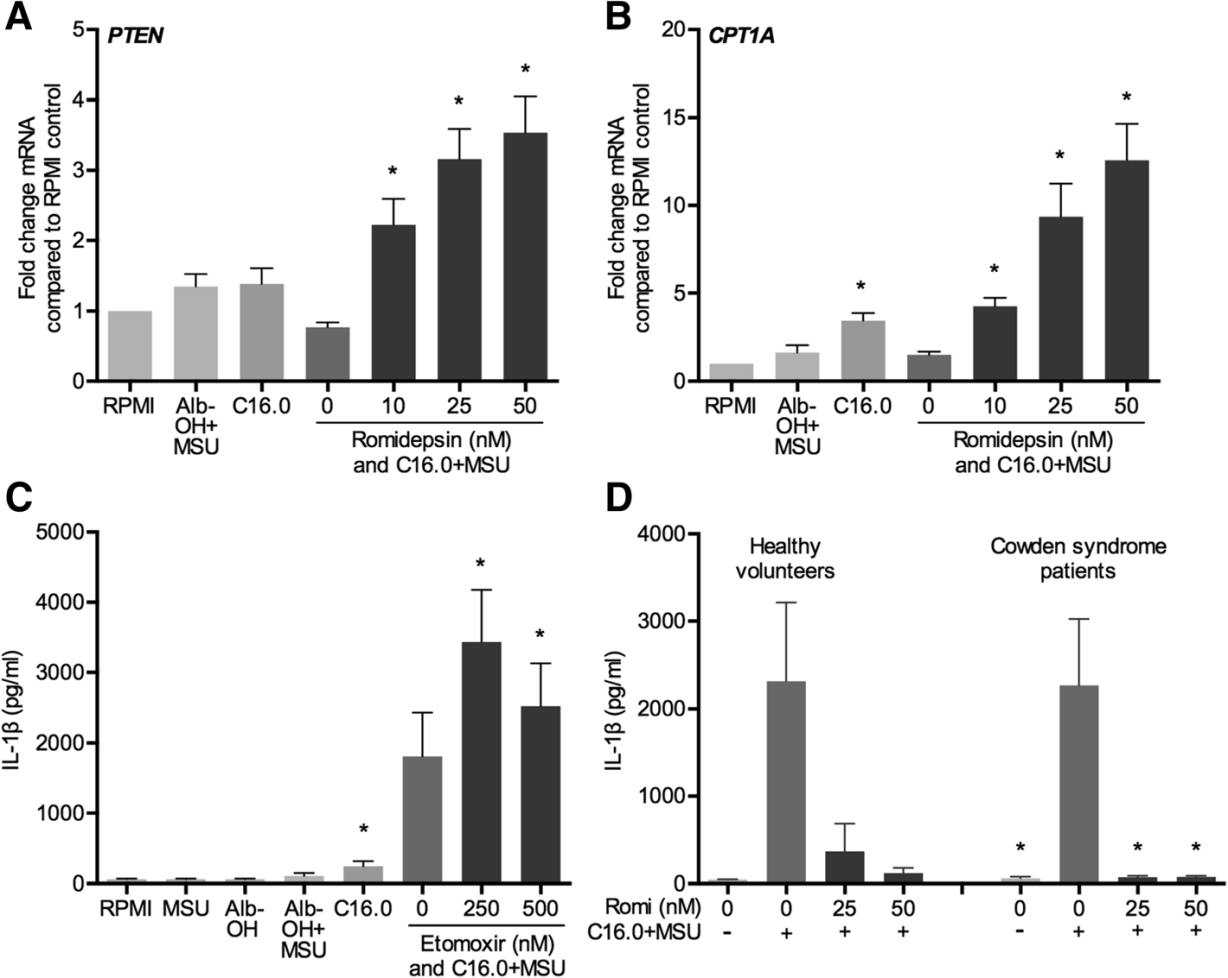
Romidepsin-induced increased expression of PTEN and CPT1A is independent of cytokine suppression. PBMCs from healthy volunteers or Cowden syndrome patients were pre-incubated for 1 h with several concentrations of romidepsin (Romi) or etomoxir. Cytokine production was induced by adding a combination of 50 μM palmitic acid (C16.0) and 300 μg/mL monosodium urate crystals (MSU). After 24 h, PTEN (**a**) and CPT1A (**b**) mRNA expression was determined by qPCR. IL-1β production was measured by ELISA after addition of etomoxir (**c**) or romidepsin in healthy volunteers and Cowden syndrome patients (**d**)

In the context of cancer, many research groups have associated HDAC inhibition with the upregulation of tumor suppressor phosphatase and tensin homolog (PTEN) and subsequent inhibition of the phosphatidylinositol 3-kinase (PI3K)/protein kinase B (Akt) pathway [19–22]. As this pathway can play an important role in cellular metabolic and inflammatory status [23–25], we assessed whether romidepsin affected PTEN expression levels. As shown in Fig. 4a, romidepsin significantly increased PTEN expression. In addition, mRNA levels of carnitine palmitoyltransferase IA (CPT1A) were also significantly elevated by romidepsin (Fig. 4b). CPT1A shuttles long-chain fatty acids, such as C16.0, into the mitochondria, comprising the rate-limiting step in the process of fatty acid oxidation. This process can be in turn regulated via the Akt signaling pathway [26, 27]. Pre-incubating the cells with etomoxir, an irreversible CPT1 inhibitor which inhibits fatty acid oxidation increased the IL-1β production in response to C16.0+MSU (Fig. 4c). To assess if PTEN upregulation mediates the cytokine-suppressive effects of romidepsin, we compared its effects in PBMCs from healthy individuals to the effects in PBMCs isolated from Cowden syndrome patients, who have a loss of function in the PTEN protein due to germline mutations. Loss of function in PTEN, however, did not reverse IL-1β suppression by romidepsin.

### Romidepsin induced SOCS1 expression and inhibited activation of STAT1 and STAT3

**Fig. 5:**
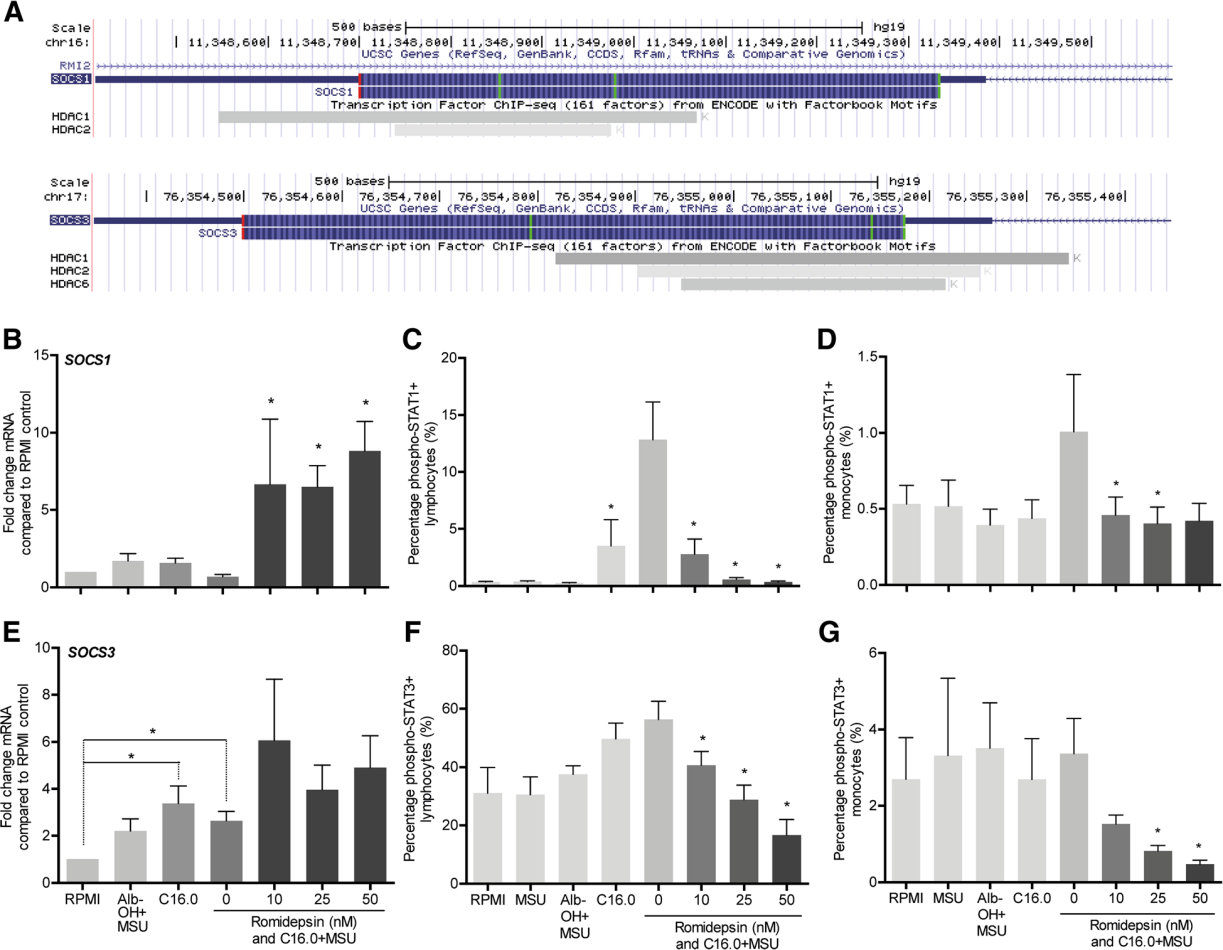
Romidepsin induced transcription of *SOCS1* and inhibited activation of STAT1 and STAT3. Schematic representations of the SOCS1 and SOCS3 genome and their binding sites for class I HDACs were retrieved from the USCS Genome Browser by means of the track “Transcription Factor ChIP-seq (161 factors) from ENCODE with Factorbook Motifs” (**a**). PBMCs from healthy volunteers were pre-incubated with romidepsin, after which a combination of 50 μM palmitic acid (C16.0) and 300 μg/mL monosodium urate crystals (MSU) was added. After 24 h, mRNA was isolated and qPCR was performed for SOCS1 and SOCS3 (**b**, **e**). Cells were stained extracellularly with CD45-KO and intracellularly with either p-STAT1-PE (**c**, **d**) or p-STAT3-PE (**f, g**) for flow cytometry. Gating on CD45+ lymphocytes and monocytes was performed in the forward versus side scatter graph

As HDAC1 and HDAC2 act primarily by deacetylating histones in the nucleus, thereby leading to hyperacetylated accessible chromatin, we envisaged that the cytokine-suppressive effects may be mediated through upregulation of anti-inflammatory genes. Suppressor of cytokine signaling (SOCS)1 and SOCS3 are important negative regulators of inflammation, and their genetic codes both include binding sites for HDAC1 and HDAC2 (Fig. 5a). Romidepsin effectively upregulated expression of SOCS1 (Fig. 5b), but not that of SOCS3 (Fig. 5e). Furthermore, romidepsin inhibited activation of the inflammatory transcription factors signal transducer and activator of transcription (STAT)1 (Fig. 5c, d) and STAT3 (Fig. 5f, g).

### The proteasome inhibitor bortezomib reverses romidepsin-induced cytokine-suppression

**Fig. 6:**
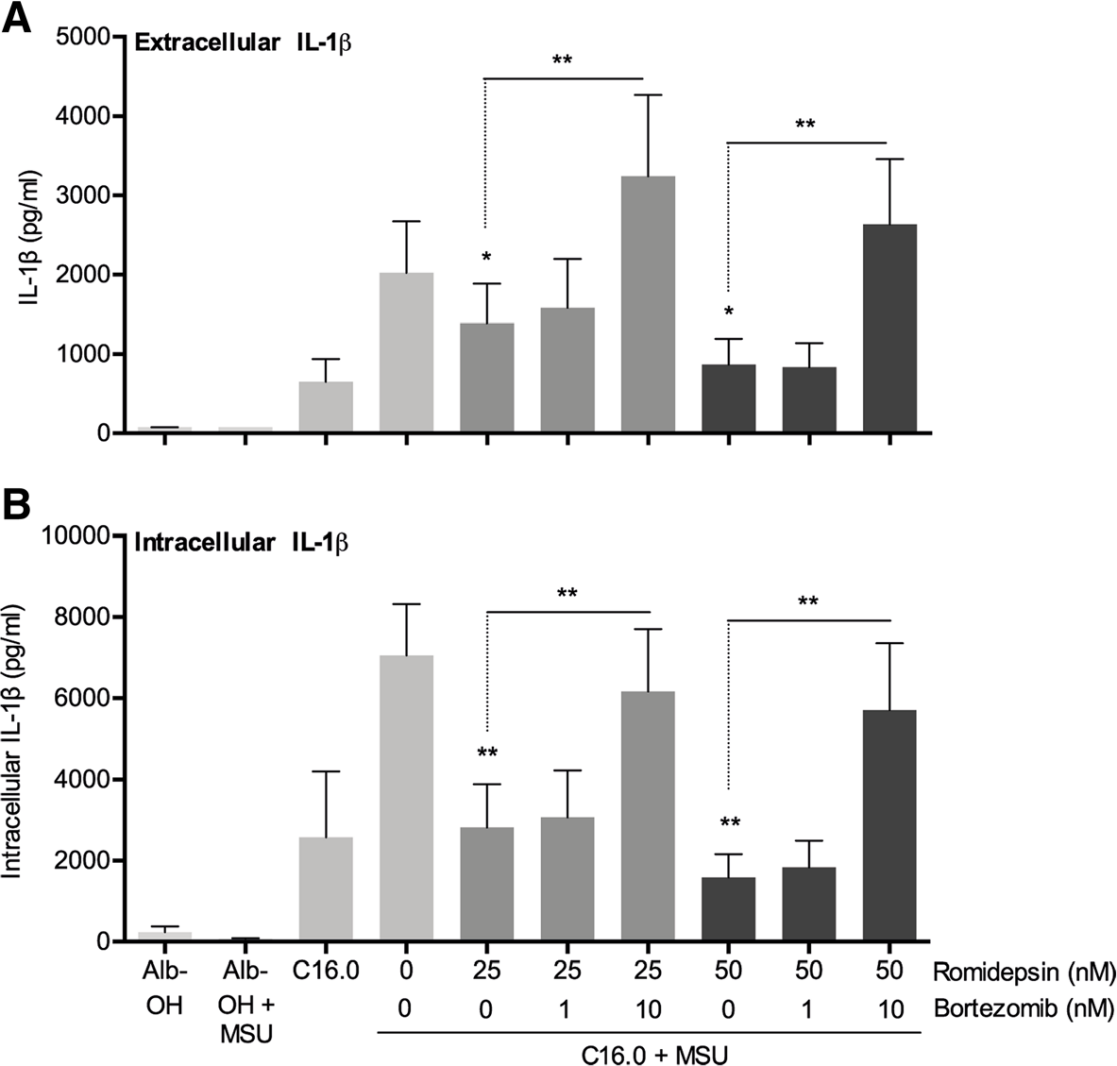
Proteasome inhibitor bortezomib reverses cytokine suppression by romidepsin. PBMCs were isolated from healthy volunteers and were pre-incubated for 1 h with bortezomib. Then romidepsin was added for 1 h pre-incubation, after which a combination of 50 μM palmitic acid (C16.0) and 300 μg/mL monosodium urate crystals (MSU) was added for another 24 h. The supernatant was collected for extracellular IL-1β (**a**). Cells were lysed with 0.5% Triton X-100, and subsequently, the supernatant of the lysate was collected for intracellular IL-1β measurement (**b**)

SOCS1 is known to induce degradation of inflammatory signaling molecules through the ubiquitin-proteasome pathway. In particular, it has been shown to induce proteasomal degradation of JAK2, p65, and TIRAP, which makes it an important negative regulator of TLR signaling pathways [28–30]. The inhibition of cytokine production by romidepsin could therefore be induced through proteasomal degradation. In Fig. 6, we show that the proteasome inhibitor bortezomib reverses the romidepsin-induced suppression IL-1β.

## Discussion

In this study, we assessed the effects of several specific HDAC inhibitors in order to pinpoint the HDACs that could play a role in acute gouty arthritis. In contrast to the use of HDAC inhibitors in cancer treatment, the anti-inflammatory effects of HDAC inhibitors are observed at very low concentrations [31]. The HDAC inhibitors givinostat (ITF2357), suberoylanilide hydroxamic acid (SAHA), and trichostatin-A have been shown to inhibit joint swelling and cell influx in several different animal models of arthritis [32–34]. More importantly, oral administration of givinostat was well-tolerated and was shown to reduce disease activity in patients with juvenile idiopathic arthritis [35].

However, most of the natural and synthetic HDAC inhibitors are broad-acting, blocking most of the classical HDACs with varying affinity. This may result in opposing effects on the immune system and cytokine production or lead to unwanted side effects. To move forward in the field of HDAC research, it is therefore important to elucidate the effects of the individual HDACs and to develop safe and orally active specific HDAC inhibitors [36, 37].

In the current study, we made use of several specific synthetic HDAC inhibitors rather than HDAC gene knockdown for several reasons. Firstly, full genetic knockout of HDAC1 or HDAC2 in mice has been shown to be lethal [38, 39]. For HDAC1- or HDAC2-specific genetic knock-down, we would have to use cell lines. This is much further from the in vivo situation in humans than we would like to be. Furthermore, expression patterns of HDACs are highly tissue-specific [40], which we envisage will lead to differential effects of HDAC inhibition in other cell types. Secondly, knockdown generally does not lead to a 100% inhibition. The synthetic HDAC inhibitors we use in this study are very potent and more likely induce HDAC inhibition to a larger extent than could be achieved by knockdown.

Here, we identify the simultaneous inhibition of HDAC1 and HDAC2 as a possible new treatment option in acute gouty arthritis. In previous experiments, we have shown that class I HDAC inhibition is effective in suppressing MSU-induced cytokine production. In addition, we showed that specific HDAC8 inhibition had no effect [11], leaving HDAC1–3 as possible mediators of the observed cytokine-suppressive effects. As a single HDAC enzyme can elicit a wide range of cellular effects, narrowing down on a specific HDAC to inhibit could be important to limit side effects. To do this, we tested several specific HDAC inhibitors. We observed that romidepsin (HDAC1/2 inhibitor) and entinostat (HDAC1/3 inhibitor) effectively reduced the production of MSU+C16.0-induced IL-1β. In contrast, single inhibition of HDAC2 or HDAC3 did not affect IL-1β levels. Finally, we tested a specific HDAC6 inhibitor. Although this is not a class I HDAC, it has been shown to associate with microtubules and could interfere with NLRP3 inflammasome assembly [41]. Our finding that it does not affect IL-1β production is in line with a previous paper showing that the HDAC6 inhibitor tubastatin did not induce migration of ASC on mitochondria towards NRLP3 on the endoplasmic reticulum [42]. Altogether, these data suggest that HDAC1 inhibition is capable of blocking IL-1β production. Due to the more potent effect of romidepsin compared to entinostat, we decided to continue with romidepsin alone.

At a concentration as low as 10 nM, romidepsin already reduced C16.0+MSU-induced inflammatory cytokines. Furthermore, intracellular levels of IL-1β and IL-1Ra, as well as IL-1β mRNA levels were decreased. We ruled out the possibility of cytokine suppression due to cell death by means of flow cytometry with Annexin V and propidium iodide staining. The slight increase in late apoptotic cells with 50 nM of romidepsin is likely caused by early apoptotic cells dying, as there is no decrease in the percentage of live cells. Although the transcriptional levels of NLRP3, CASP1, and PYCARD seemed to be affected by romidepsin, the effects are small and do not follow the striking dose-response as is seen in the cytokine production. There could still be post-transcriptional changes in the activity of the NLRP3 inflammasome. However, we envisage that this is unlikely, as we see no accumulation of intracellular IL-1β upon addition of romidepsin, and because HDAC1 and HDAC2 mostly affect transcriptional activity in the nucleus. These results suggest that romidepsin blocks IL-1β production at the level of pro-IL-1β transcription.

A likely candidate to mediate this effect would be the NF-κB transcription factor. In the canonical pathway, activation of the RelA protein is required to induce pro-inflammatory gene transcription. However, several studies have shown that acetylation of RelA in fact causes increased activation of NF-κB [43–46]. Inhibition of HDACs would therefore lead to activation of this transcription factor. This does not fit with the significant decrease in cytokine production upon addition of romidepsin. Therefore, this pathway was not explored further within the scope of this manuscript.

In the context of cancer, romidepsin has been shown to interfere with the PTEN/PI3K/Akt pathway [19–22]. This pathway plays a key role in the control of autophagy and cellular metabolism and can indirectly affect the inflammatory status of the cell [25, 47]. In the current study, romidepsin indeed increased expression of *PTEN,* and also of *CPT1A*, suggesting a decreased activation of the PI3K/Akt pathway. However, in the absence of functional PTEN in Cowden syndrome patients, romidepsin still potently suppressed IL-1β production, indicating that the cytokine-suppressive effects of romidepsin are independent of PTEN.

In our search for a possible mechanism of romidepsin-mediated cytokine suppression, we found that both *SOCS1* and *SOCS3* genes possess a binding site for HDAC1 and HDAC2. Subsequently, we showed that romidepsin significantly increases gene expression of *SOCS1*, but not of *SOCS3*. In addition, a significant decrease in STAT1 and STAT3 phosphorylation was observed in both monocytes and lymphocytes. Naturally, the decreased activation of STAT1 could be the result of SOCS1 upregulation. The decrease in STAT3 activation, however, is independent of SOCS3 and may be mediated via a romidepsin-induced decrease in IL-6 production. Several research groups recently established an interesting direct link between SOCS1 and IL-1β. This link involves targeting several inflammatory signaling proteins for proteasomal degradation [28–30]. To test this pathway, we studied the effect of proteasome inhibitor bortezomib on romidepsin-induced cytokine suppression. We found that indeed bortezomib was able to reverse the suppression of IL-1β by romidepsin, indicating that proteasomal degradation is a key mechanism by which romidepsin exerts its effects.

Romidepsin (Istodax®) was FDA-approved in 2009 for treatment of cutaneous T cell lymphoma and is administered intravenously at 14 mg/m2 over a 4-h period on days 1, 8, and 15 of a 28-day cycle [48]. We envisage that a much lower concentration could be used to treat gouty arthritis. Future in vivo studies will have to show whether this is a potential treatment option in gouty arthritis. Due to the potent anti-inflammatory effects, HDAC inhibition may be used in the future to treat other auto-inflammatory diseases as well.

Additionally, it would be very valuable to study the effects of romidepsin mechanistically by linking acetylation events in specific genetic loci to altered inflammatory gene transcription by means of chromatin immunoprecipitation and genetic sequencing (ChIP-seq). We have not performed this in the current study due to the high costs related to such methods. Alternatively, ChIP-PCR would lack sensitivity for several reasons. Firstly, the current view that HDAC inhibitors increase histone acetylation and increase gene transcription is most likely oversimplified. Genome-wide analyses revealed increased broad deacetylation conferred by HDAC inhibitors in vascular endothelial cells, mediated by the loss of EP300/CREBBP binding [49]. Furthermore, only one target can be studied at a time with ChIP-PCR. HDACs have low substrate specificity, and there are several histone lysines known to be subject to acetylation [50]. Studying histone acetylation is further complicated by the fact that HDAC1 and HDAC2 are present together in several repressing complexes (NuRD, Sin3a and co-REST complexes). Finally, there is abundant cross-talk between different histone modifications, affecting one another [50].

## Conclusion

Taken together, we can conclude that inhibition of HDAC1 and HDAC2 by romidepsin effectively decreases C16.0+MSU-induced cytokine production. Its effects are most likely mediated via increased acetylation and subsequent increased expression of the *SOCS1* gene. SOCS1 is able to directly target inflammatory signaling molecules for proteasomal degradation, which could prevent the initial transcription of IL-1β. Although romidepsin is a very potent inhibitor in vitro, more studies are required before we can achieve HDAC1/2 inhibition during a gout flare in patients.

## Additional file

Additional file 1: **Figure S1.** Effects of romidepsin on C16.0+MSU-induced IL-10 production in human PBMCs. Freshly isolated PBMCs were pre-incubated with different concentrations of romidepsin for 1 h, after which cytokine production was induced via addition of a combination of 50 μM palmitic acid (C16.0) and 300 μg/mL monosodium urate crystals (MSU). The cells were cultured for 24 h. IL-10 concentration was measured in the supernatant. (PDF 160 kb)

## Abbreviations

Akt: Protein kinase B
AnV: Annexin V
ASC: Apoptosis-associated speck-like protein containing a CARD
CASP1: Caspase-1
cDNA: Complementary deoxyribonucleic acid
CPT1A: Carnitine palmitoyltransferase 1A
ELISA: Enzyme-linked immunosorbent assay
HDAC: Histone deacetylase (11 numbered human enzymes)
IL1b: Interleukin-1β (also interleukin-1Ra, interleukin-6, and interleukin-8)
JAK2: Janus kinase 2
MSU: Monosodium urate
NLRP3: NOD-like receptor pyrin domain-containing-3
PBMC: Peripheral blood mononuclear cell
PCR: Polymerase chain reaction
PI: Propidium iodide
PI3K: Phosphoinositide 30kinase
PTEN: Phosphatase and tensin homolog
PYCARD: Gene for apoptosis-associated speck-like protein containing a CARD
RNA: Ribonucleic acid
RPMI: Roswell Park Memorial Institute medium
SAHA: Suberoylanilide hydroxamic acid
SIRT: Sirtuin
SOCS: Suppressor of cytokine signaling (in this manuscript SOCS1 and SOCS3)
STAT: Signal transducer and activator of transcription
TIRAP: TIR-domain containing adapter protein
TLR: Toll-like receptor
