# Supplementary material for: Romidepsin suppresses monosodium urate crystal-induced cytokine production through upregulation of suppressor of cytokine signaling 1 expression
Source: Arthritis Res Ther. 2019 Feb 6;21:50. doi: 10.1186/s13075-019-1834-x (PMC6366029; doi:10.1186/s13075-019-1834-x)
Supplement: Supplementary file 1 — Figure S1. Effects of romidepsin on C16.0+MSU-induced IL-10 production in human PBMCs. Freshly isolated PBMCs were pre-incubated with different concentrations of romidepsin for 1 h, after which cytokine production was induced via addition of a combination of 50 μM palmitic acid (C16.0) and 300 μg/mL monosodium urate crystals (MSU). The cells were cultured for 24 h. IL-10 concentration was measured in the supernatant. (PDF 160 kb) [file 13075_2019_1834_MOESM1_ESM.pdf]

Supplemental data

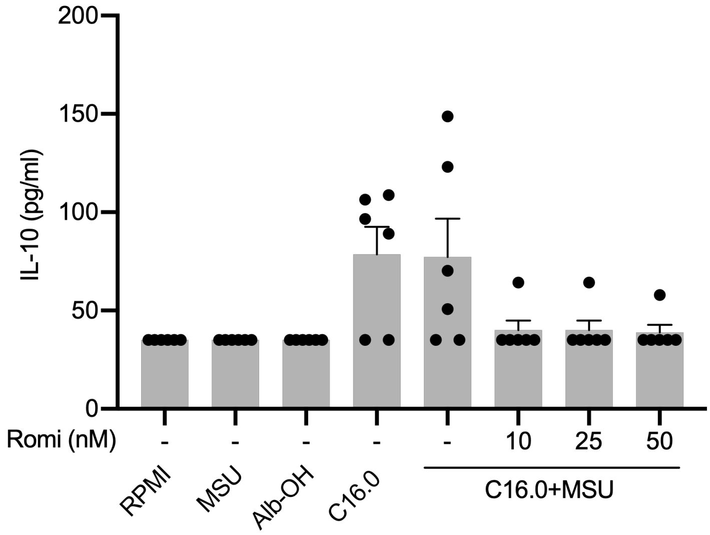

Supplemental Figure 1. Effects of Romidepsin on C16.0+MSU-induced IL-10 production in human PBMCs

Freshly isolated PBMCs were pre-incubated with different concentrations of Romidepsin for 1 hour, after which cytokine production was induced via addition of a combination of 50 µM palmitic acid (C16.0) and 300 µg/mL monosodium urate crystals (MSU). The cells were cultured for 24 hours. IL-10 concentration was measured in the supernatant.
